# Chromophobe renal cell carcinoma or oncocytoma: a manner of challenge in frozen section diagnosis

**DOI:** 10.1051/bmdcn/2019090106

**Published:** 2019-02-22

**Authors:** Farideh Baharzadeh, Masoud Sadeghi, Mazaher Ramezani

**Affiliations:** 1 Students Research Committee, Kermanshah University of Medical Sciences Kermanshah Iran; 2 Medical Biology Research Center, Kermanshah University of Medical Sciences Kermanshah Iran; 3 Molecular Pathology Research Center, Imam Reza Hospital, Kermanshah University of Medical Sciences Kermanshah Iran

**Keywords:** Chromophobe RCC, Oncocytoma, Frozen section, Case report

## Abstract

Chromophobe renal cell carcinoma (RCC) is a rare type of kidney neoplasm that is diagnosed in the 6th decade of life with similar incidence in male and female. We reported a case of 73-year-old man with a chief complaint of nocturia, frequency, dribbling and urinary retention with renal mass in ultrasound examination. Histologic examination and immunohistochemistry study revealed the diagnosis of chromophobe RCC which initially was mistaken for oncocytoma in frozen section diagnosis. The pathologist should be aware of this malignant entity and be cautious in diagnosing oncocytoma in frozen section and routine H & E staining.

## 1. Introduction

Chromophobe renal cell carcinoma (RCC) is a rare neoplasm of the kidney that represents about 5% of RCCs. This malignant neoplasm of kidney is clinically diagnosed with an earlier stage and better prognosis than conventional clear-cell RCC [1]. The 5-and 10-year survival rates of this cancer are reported 100 and 90%, respectively [2]. This neoplasm is more common in the 6th decade of life [3]. The incidence of chromophobe RCC is equal in male and female population [4, 5]. The symptoms include flank pain and mass, hematuria, weight loss, renal dysfunction, and pain from metastatic sites [6]. The purpose of the study was to report a case with chromophobe RCC that had a challenge in frozen section diagnosis. On the other words, the pathologists must be kept in mind that, encountering a renal mass with oncocytic features or eosinophilic cytoplasms in frozen section is not equal to merely oncocytoma. An important differential diagnosis of chromophobe RCC needs to be suggested to the surgeon and definite diagnosis should be postponed to permanent sections and immunohistochemistry (IHC) study.

## 2. Case report

**Fig. 1:**
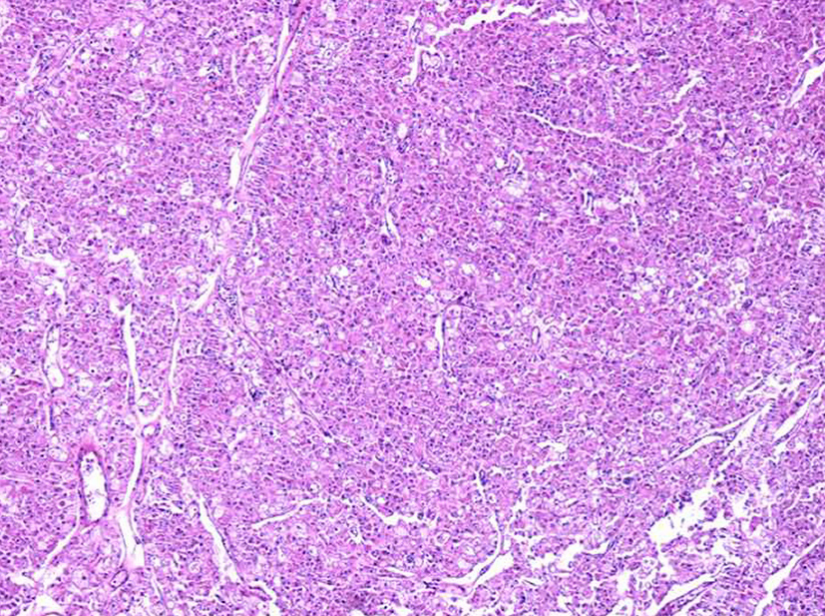
Chromophobe renal cell carcinoma mimicking oncocytoma: Permanent of frozen section, Hematoxylin & chemistry staining (×40 magnification).

**Fig. 2:**
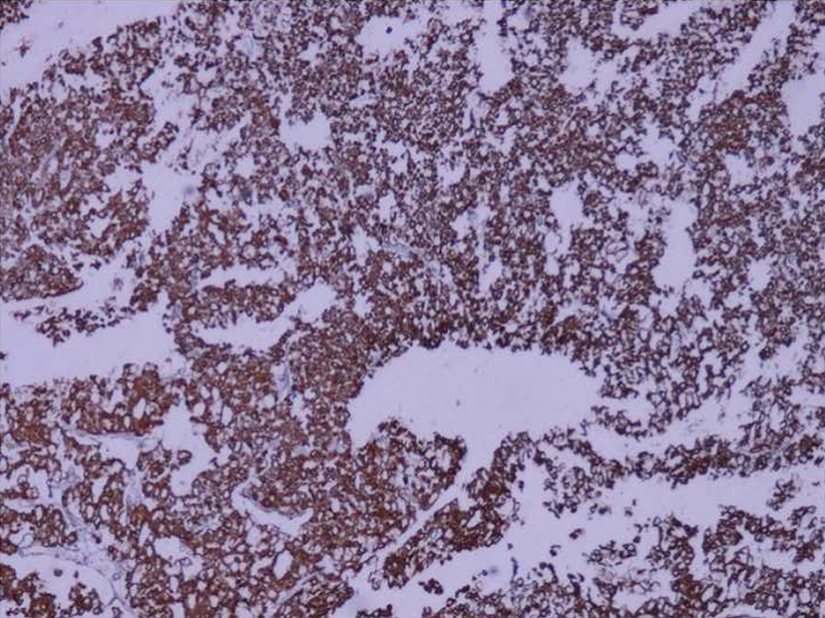
CK7-positive tumor cells: Immunohistochemistry staining (*40 magnification).

**Fig. 3:**
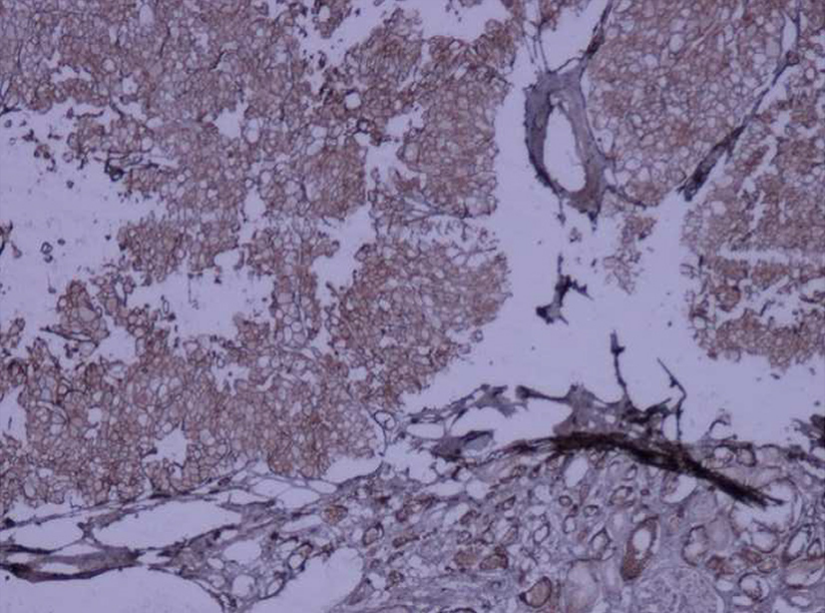
E-cadherin positivity tumor cells: Immunohistochemistry staining (×40 magnification).

**Fig. 4:**
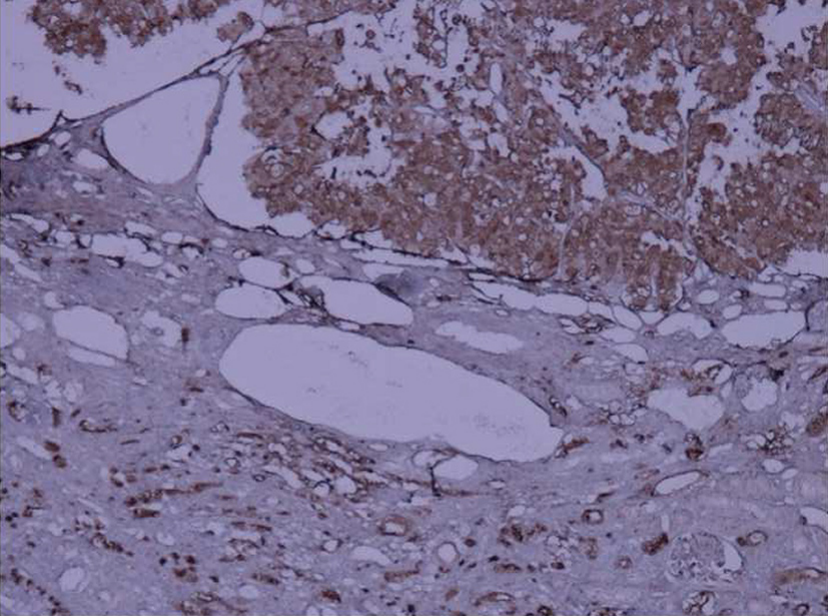
Epithelial membrane antigen positivity tumor cells: Immunohistochemistry staining (×40 magnification).

A 73-year-old man was admitted in Urology ward on 30^th^ September 2017 with a chief complaint of nocturia, frequency, dribbling, and urinary retention since last month. Ultrasound examination revealed left renal mass. In past history diabetes mellitus, hypertension, and ischemic heart disease was noted. He stopped cigarette smoking 20 years ago. His drug history was Enalapril, ASA, Metformin, and Metoral. The lab data including complete blood count, Blood Urea Nitrogen (BUN), creatinine, Na, K, Arterial Blood Gas (ABG), Prostatic Specific Antigen (PSA), and free PSA were within normal limits except for mild anemia (Hemoglobin: 10.6 gr/dl). Blood sugar (BS) was 159 mg/dl. Urine analysis showed 10-15 white blood cells (WBC) in high power field with a negative urine culture. In digital rectal examination prostate was nodular (2-3+) and symmetric. Ultrasound examination on 7^th^ October 2017 demonstrated mild bilateral hydroureteronephrosis with the over distended urinary bladder. Prostatic volume was 25 cc with the retained urine of 950 cc. A well-defined hypoechoic exophytic mass without calcification measuring 60 × 38 mm in left renal pole with vascular areas was noted. Computer tomography scanning on 10^th^ October 2017 revealed 44 × 38 mm hypo attenuated mass in the lower pole of the left kidney with arterial and portal enhancement and delay washout in favor of infiltrative process such as RCC close to left psoas muscle. Mild bilateral hydronephrosis due to enlarged prostate was seen. No lymphade- nopathy in pelvis and abdomen was seen. He referred to surgery department of the hospital for nephrectomy. The specimen was referred to the pathology department. The frozen section microscopic evaluation revealed oncocytic feature in favor of oncocytoma. In permanent diagnosis, a portion of renal tissue measuring 6.5 × 5.5 × 3.5 cm with perirenal fatty tissue measuring 3 × 2 × 1 cm was evaluated. In cut section, creamy brown solid mass measuring up to 5.5 cm at 1.5 cm distance from renal resected margin was noted. The pathologist reported chromophobe RCC (Fig. 1) with vascular and renal capsule invasion but with no necrosis or margin involvement. The IHC was done and tumor cells were positive for cytokeratin (CK) 7, CK8, CK20 (weakly +), the Epithelial Membrane Antigen (EMA), Cluster of Differentiation (CD) 10, E-Cadherin and High Molecular Weight Keratin (HMWK, focally positive). Inhibin and vimentin markers were negative in tumor cells (Fig. 2–4). The findings were in favor of chromophobe RCC. We followed- up the patient for 5 months after surgery. No recurrence or metastasis was diagnosed.

## 3. Discussion

Chromophobe RCC is a type of renal neoplasm that first described in 1986 [7, 8]. Chromophobe RCC is more common in 6^th^ decade of life with the similar incidence in males and females (4, 5). This neoplasm is usually diagnosed in stage I and II [9]. The most common clinical symptoms are hematuria, flank mass and pain [10]. Chromophobe RCC is usually a hypodense mass in CT scan with homogeneous enhancement. Calcification is seen in 38% of the cases [11]. Metastasis is present in about 6% of RCC cases with most frequency in liver and lung. Renal vein invasion is found in less than 5% of all RCC cases [12].

Chromophobe RCC is a rare subtype of RCC with different histochemical, ultra structural, and genetic features [12, 13]. The neoplasm originates from the intercalated cells of the collecting ducts [14]. In the gross examination, this tumor is solitary, circumscribed and not capsulated mass [15]. Three variants of chromophobe RCC are known. First, the classic variant with more than 80% pale cells that this variant is associated with necrosis and sarcomatoid features with high growth rate and metastasis and second, the eosinophilic variant with more than 80% eosinophilic cells. The histologic picture is similar to oncocytomas. The third variant is mixed [4]. The classic variant is composed of well-defined cells with wrinkled nuclei and abundant pale granular cytoplasm (the type III cell). This type has diffuse reticular cytoplasmic staining with Hale’s colloidal iron. The eosinophilic variant is less frequent and is composed of smaller cells with eosinophilic granular cytoplasm (the type I cells). The type II cells are similar to the type I cell, but are larger with perinuclear translucent zone. The eosinophilic variant may be mistaken for oncocytoma. Histologic features in favor of eosinophilic variant chromophobe RCC are the sheet-like arrangement, wrinkled nuclei and the presence of type II and type III cells along with type I cells. Oncocytoma has nested and tubular pattern with round hyperchromatic nuclei and degenerative atypia. Hale’s colloidal iron shows focal positivity in the luminal borders of cytoplasms in oncocytoma [6, 16].

Tumor cells show a strong positive reactivity for CK7 and EMA with negative reaction for CD10 and vimentin in IHC study, although the reported case was CD10 positive [10, 11, 17]. These tumors are also positive for CD117 (c-kit) [18, 19]. Surgery is the main treatment for chromophobe RCC. Chemotherapy is not an effective treatment for advanced chromophobe RCC [20, 21]. The prognosis of chromophobe RCC is better than conventional RCC, even in metastatic disease. Distant metastases are more found in liver and lungs. The studies proved that patients with chromophobe RCC have a good prognosis and survival rates in early stage [20].

In conclusion, chromophobe RCC may mimic oncocytoma on frozen section study and Hematoxylin & Eosin staining, and also investigators have encountered difficulty in distinguishing it from oncocytoma histologically. IHC has a benefit for differentiation. The importance of distinguishing Chromophobe RCC from oncocytoma is because of the different prognosis.

## 4. Conclusion

Chromophobe renal cell carcinoma, mainly eosinophilic variant, may show oncocytic features and mimicking oncocytoma in frozen section and routine histopathology specimens. Immuno- histochemistry panel, including CK7, CK8, CK20, EMA, CD10, E-Cadherin, HMWK, Inhibin, Vimentin, and CD117 is suggested for definite diagnosis. Pathologists must be aware of this pitfall and avoid definite diagnosis of Chromophobe renal cell carcinoma or oncocytoma especially in frozen section session without immunohistochemistry panel.
